# Verbal/social autopsy study helps explain the lack of decrease in neonatal mortality in Niger, 2007–2010

**DOI:** 10.7189/jogh.06.010604

**Published:** 2016-06

**Authors:** Henry D Kalter, Asma Gali Yaroh, Abdou Maina, Alain K Koffi, Khaled Bensaïd, Agbessi Amouzou, Robert E Black

**Affiliations:** 1 Department of International Health, Johns Hopkins Bloomberg School of Public Health, Baltimore, MD, USA; 2 Ministry of Health, Niamey, Niger; 3 Institute National des Statistics, Niamey, Niger; 4 UNICEF, Niger country office, Niamey, Niger (retired staff); 5 UNICEF, New York City, NY, USA; 6 The Institute for International Programs, Johns Hopkins Bloomberg School of Public Health, Baltimore, MD, USA

## Abstract

**Background:**

This study was one of a set of verbal/social autopsy (VASA) investigations undertaken by the WHO/UNICEF–supported Child Health Epidemiology Reference Group to estimate the causes and determinants of neonatal and child deaths in high priority countries. The study objective was to help explain the lack of decrease in neonatal mortality in Niger from 2007 to 2010, a period during which child mortality was decreasing.

**Methods:**

VASA interviews were conducted of a random sample of 453 neonatal deaths identified by the 2010 Niger National Mortality Survey (NNMS). Causes of death were determined by expert algorithm analysis, and the prevalence of household, community and health system determinants were examined along the continuum of maternal and newborn care, the Pathway to Survival for newborn illnesses, and an extended pathway for maternal complications. The social autopsy findings were compared to available data for survivors from the same cohort collected by the NNMS and the 2012 Niger Demographic and Health Survey.

**Findings:**

Severe neonatal infection and birth asphyxia were the leading causes of early neonatal death in the community and facilities. Death in the community after delayed careseeking for severe infection predominated during the late neonatal period. The levels of nearly all demographic, antenatal and delivery care factors were in the direction of risk for the VASA study decedents. They more often resided rurally (*P* < 0.001) and their mothers were less educated (*P* = 0.03) and gave birth when younger (*P* = 0.03) than survivors’ mothers. Their mothers also were less likely to receive quality antenatal care (*P* < 0.001), skilled attendance at birth (*P* = 0.03) or to deliver in an institution (*P* < 0.001). Nearly half suffered an obstetric complication, with more maternal infection (17.9% vs 0.2%), antepartum hemorrhage (12.5% vs 0.5%) and eclampsia/preeclampsia (9.5% vs 1.6%) than for all births in Niger. Their mothers also were unlikely to seek health care for their own complications (37% to 42%) as well as for the newborn’s illness (30.6%).

**Conclusions:**

Niger should scale up its recently implemented package of high–impact interventions to additional integrated health facilities and expand the package to provide antenatal care and management of labor and delivery, with support to reach a higher level facility when required. Community interventions are needed to improve illness recognition and careseeking for severe neonatal infection.

The 2010 Niger National Mortality Survey (NNMS) found that from 1998 to 2009 the mortality rate of children less than 5 years old decreased significantly by 43.4%, from 226 (95% confidence interval CI 207–246) to 128 (95% CI 117–140) deaths per 1000 live births, but mortality of neonates less than 28 days old declined insignificantly from 39 (95% CI 32–46) to 33 (95% CI 28–39) deaths per 1000 live births [1,2]. The reduction in child deaths was attributed to improvements in the nutritional status of children less than 2 years old and increased coverage of key child survival interventions, including insecticide–treated bed nets, vitamin A supplementation, treatment of diarrhea with oral rehydration salts and zinc, careseeking for childhood pneumonia and fever or cough, and vaccinations. The rapid uptake of interventions was achieved through government policy decisions to implement the Integrated Management of Childhood Illness (IMCI) approach, integrated community case management for children with fever or malaria, suspected pneumonia and diarrhea, and to provide free health care for all pregnant women and children including scaling up access to a minimum package of high–impact interventions at integrated health centers and health posts.

Interventions effective against neonatal mortality that were examined, including antenatal care, maternal tetanus toxoid, skilled birth attendance, early initiation of and exclusive breastfeeding, showed smaller increases in coverage to endpoint levels well below 50%, likely inadequate to decrease neonatal mortality [1]. In addition, an earlier study on the quality of maternal and newborn care found that few health workers present at birth had the knowledge, skills and access to basic equipment needed to effectively manage obstetric and newborn problems. Only 2.5% of Centres de Santé Intégrés (CSI), which are meant to have at least two nurses or midwives on duty at all times and which are the main health centers throughout the country intended to provide Basic Emergency Obstetric and Neonatal Care (BEmONC), had the full capacity for this service; and the national met need for EmONC stood at 2.3%, varying by region from 1.4% to 6.5% [3]. Health posts (Case de Santés), only about one–fourth of which have a nurse or midwife on staff and are not intended to provide EmONC, were not examined.

Neither of these studies, however, examined several other interventions critical to neonatal survival nor did they assess the causes of and events leading up to the deaths of the newborns along the continuum of antenatal and delivery care of the mother and immediate postnatal care of the newborn, maternal complications and the severe newborn illnesses these can lead to, mothers’ perceptions and knowledge of how to respond to such critical events, their careseeking attempts for themselves and their newborns, and factors affecting these behaviors.

The fact that maternal complications occur at a fairly constant level, severe enough to kill the mother in about 1.0% to 1.4% of pregnancies and to kill the baby at a much higher rate, and that it cannot be reliably predicted which women will experience these complications, is the basis for the maternal mortality reduction strategy of universal access to skilled birth attendance and emergency obstetric care when needed [4–7]. This strategy is no less important to the survival and health of the neonate, as it has been shown that pregnancy and delivery complications are the most important risk factors for neonatal mortality [8–12], with care directed at the intrapartum period providing the greatest mortality reduction [13]. Integrated maternal–neonatal care packages and linkages of community with facility maternal and newborn care provide further reductions in stillbirths and neonatal deaths [14,15]. The addition of newborn–specific strategies, including fetal monitoring, access to Caesarean section for fetal distress, clean delivery and cord care, neonatal resuscitation, early initiation of and exclusive breastfeeding, timely and appropriate thermal care of the baby, kangaroo mother care for stabilized preterm infants, recognition of and early careseeking for newborn illness, access to quality health care, and urgent referral to neonatal intensive care when needed, are required to maximize newborn survival [13,16–20].

Examining such vital information on maternal and newborn care provided for babies that died is needed to help explain why the deaths occurred and how they might have been prevented. Collecting comparison data for newborns that suffered a severe but non–fatal illness during the same time period as the deaths would require the difficult task of identifying households where such an illness occurred; and the inability to appropriately match deaths with other cases on the basis of illness severity and the timing of clinical signs has led to a misleading situation where one could falsely conclude that treatment increased mortality risk [21]. Moreover, promoting neonatal health and preventing the death of sick newborns requires well–proven interventions for which the population levels established by already–completed surveys can provide reasonable comparisons for the surveyed factors.

Social autopsy (SA) is a method of inquiring about deaths that adds questions on household, community and health system determinants of mortality to complement a verbal autopsy (VA) interview on the illness signs and symptoms used to establish the biological cause of death [22]. We undertook to assess the biological causes and social determinants of recent neonatal deaths in Niger by conducting a verbal/social autopsy (VASA) study of neonatal deaths that occurred in 2007–2010 and were identified by the 2010 NNMS. Where possible, we compared the VASA findings for the deaths to the same factors for surviving children from the same cohort determined by recent population surveys. In this way, we sought to further explain the reasons for the limited decrease in neonatal mortality in Niger from 2007 to 2010.

## METHODS

The VASA was a descriptive study of the causes of death and the prevalence of key determinants of a national random sample of neonatal deaths derived from the 2010 NNMS’s full birth history interview of women aged 15–49. Where possible, the levels of key determinants for the decedents were compared to the same factors for surviving children from the same cohort determined by the 2010 NNMS and the 2012 Niger Demographic and Health survey (NDHS); and maternal complications for surviving children were ascertained by the 2010–2011 WHO Multicountry Survey on Maternal and Newborn Health [12].

### Data

The study sample has been fully described elsewhere [23]. In brief, the deaths included in the VASA study were identified by the lifetime birth history interview conducted of all women 15 to 49 years old who participated in the 2010 NNMS [24]. The VASA study considered only the 2380 under 5 years old (734 neonatal, 0 to 27 days old and 1646 child, 1 to 59 months old) deaths as far back from the survey period as four years. From these, in order to minimize the interview recall period, we started with the most recent death and moved backwards, taking the one most recent death in each household with at least one under 5 years old death until the desired sample sizes of 605 neonatal and 605 child deaths had been achieved.

The final VASA sample consisted of 1166 (96.9%) completed interviews of 1203 attempted, including 453 neonatal deaths, 620 child deaths and 93 stillbirths. Although the NNMS was designed to identify only live births and child deaths, some survey–classified (mainly) neonatal deaths were determined by the more detailed VASA interview to have been stillbirths, as defined by the caregiver’s report that the child was born dead and never cried, breathed or moved. These discrepancies, as well as some movement between the neonatal and child categories, were checked during revisits to the households in question. The final VASA–determined birth status and age at death were taken as the correct data for this study. This paper examines the 453 neonatal deaths.

### VASA interview

The VASA questionnaire, its translation, and the study’s interview methods also have been fully described [23]. To sum up, the questionnaire blends the Population Health Metrics Research Consortium (PHMRC) VA questionnaire [25] with the Child Health Epidemiology Reference Group (CHERG) SA questionnaire [22]. The interviews were conducted in French and the two main languages of Niger, Haoussa and Zarma, using a CSProX [26] software application developed for the VASA study to assist interviewers to capture responses with minimal data entry errors in the field directly on netbook computers. Most of the fieldwork was conducted from March–April 2012. Revisits to some households extended the data collection until September 2012.

The interviewers were 12 female and eight male native speakers of Haoussa and/or Zarma, all secondary school graduates and 86% with some post–secondary education. They received 10 days of classroom training in all aspects of the VASA study and three days of field practice in conducting the interview. The seven teams, each with its interviewers and one supervisor, completed the data collection in 55 days. The respondent was the person most closely involved in caring for the child during the fatal illness, which typically is the mother. Secondary respondents were allowed, if necessary, to capture information on all phases of the illness, including the mother’s pregnancy and delivery, during which she may herself have been ill and so less aware of the child’s condition. In case of any disagreement the main respondent’s answer was always taken as final.

### Neonatal cause of death assignment

Verbal autopsy algorithms arranged in a hierarchy were used to assign the main cause of death for each neonate. The development of the algorithms and hierarchy and analysis to determine the causes of death also have been fully described [23]. Briefly, the algorithms were based on prior validation studies, additional verbal autopsy expert consultation, a literature review to identify illness signs and symptoms associated with particular neonatal illnesses, and the development of new algorithms for previously non–validated conditions. The hierarchy was developed to select the main, usually underlying, cause of death among all co–morbid conditions identified by the algorithms.

### Social determinants of neonatal death

The Pathway to Survival [27] conceptual model was used to organize the collection and analysis of the social autopsy data on the health promotive, disease preventive and curative actions taken for children inside– and outside–the–home. An extended pathway for neonatal survival was developed to examine the mothers’ antenatal and delivery care, pregnancy and delivery complications and careseeking for these. All the indicators examined along the pathways are of proven interventions against neonatal mortality contained in the Lives Saved (LiST) tool [28], judged by an evidence review or recommended by the WHO. The VASA study also assessed factors that might help explain why desirable actions were not taken, including socio–economic and demographic factors, recognition of illness severity, who were the decision makers, and self–identified careseeking constraining factors.

### Illness severity

Caregivers’ reports of their child’s symptoms indicating severe or possibly severe illness at the time of illness onset were used to evaluate the appropriateness of the first action taken in response to the illness. Symptoms’ severity was rated according to their use in the Integrated Management of Childhood Illness (IMCI) approach [29], with any symptom signifying the need for urgent referral being ranked as “severe” and other symptoms yielding an IMCI disease classification requiring treatment ranked as “possibly severe.” The severity of symptoms included in the verbal autopsy but not in the IMCI was rated by two of the study authors (HDK and AKK, both physicians). In addition to the rating of individual symptoms’ severity, illness severity syndromes of symptoms recognized by mothers as indicating the need for health care [30-33] were formed by combining caregivers’ reports of their child’s feeding behavior, alertness and activity level at key points during the illness and used to evaluate the appropriateness of actions taken at those times. The method used to rank the severity of the syndromes has been previously described [34].

### Maternal complications

As part of the extended pathway for neonatal survival, the presence of and careseeking for seven pregnancy and seven delivery complications were assessed. Because there is much overlap of the symptoms between major obstetrical conditions that can lead to over counting of complications, we examined the complications as defined by the symptom syndromes displayed in **Box 1**.

**Figure 1:**
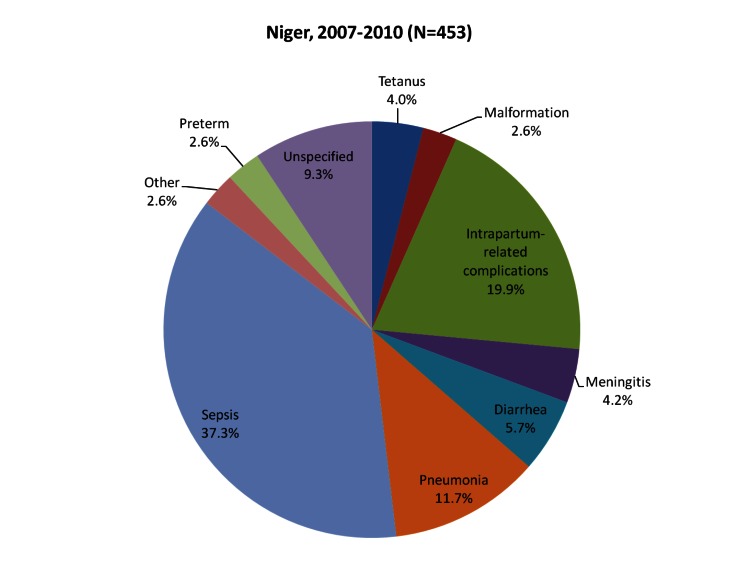
Expert algorithm, hierarchical verbal autopsy causes of death for 453 neonatal deaths, Niger, 2007–2010.

### Comparison data

The 2010 NNMS was examined for data on factors comparable to those included in the VASA study for surviving children who were their mother’s most recent birth in 2007–2010 (**Table 1** and **Table 2**). The NNMS collected data on maternal care variables only for births in the 12 months prior to the survey. Some variables not assessed by the 2010 NNMS were available from the 2012 NDHS [35]. These data also were examined for surviving children who were their mother’s last birth in 2007–2010 (**Table 2**). **Table 3** displays baseline levels of maternal complications prior to surviving births established by the 2010–2011 WHO Multicountry Survey on Maternal and Newborn Health [12], including 10 871 births in Niger.

**Table 1:** Comparison of demographic characteristics of 453 neonatal deaths with those of the general population, Niger, 2007–2010

	Neonatal deaths			Comparison data*				
**Characteristic**	**N**	**%, mean or median**	**(Q1, Q3)**	**N**	**%, mean or median**	**(Q1,Q3)**	**χ^2^**	***P*–value**
**Sex:**								
Male	263	58.1		8360	50.8		9.3	0.002
Female	190	41.9		8082	49.2			
**Birth order:**								
1	114	25.3		2317	14.1		44.3	<0.001
2–3	92	20.4		4878	29.7			
4+	245	54.3		9212	56.2			
**Mother’s age at first marriage:†**								
Median age (years)	425	15.0	(15, 17)	8938	15.0	(14, 17)		
**Mother’s age at birth of index child:**								
<20	101	22.8		2559	15.7		16.3	<0.001
20–24	121	27.2		4112	25.2			
25–29	97	22.0		4302	26.4			
30+	124	28.0		5338	32.7			
Mean age (years)	443	25.6	(20, 31)	16 312	26.9	(21, 31)		
**Mother’s age at first birth:**								
<15	41	9.2		1190	7.4			
15–19	273	61.8		9400	58.8			
20+	128	29.0		5409	33.8		4.5	0.034
Mean age (years)	442	18.4	(16, 20)	15 999	18.7	(16, 20)		
**Mother’s education:**								
None	386	86.6		13 521	82.7		4.7	0.030
Primary	47	10.6		1890	11.6			
Secondary+	13	2.9		948	5.8			
Median years	446	0.0	(0, 0)	16 359	1.0	(1, 2)		
**Father’s education:**								
None	369	84.7		–	–			
Primary	46	10.6		–	–			
Secondary+	20	4.7		–	–			
Median years	434	0.0	(0, 0)	–	–			
**Residence:**								
Urban	44	9.7		2994	18.2			
Rural	409	90.3		13 450	81.8		21.5	<0.001
**Travel time (min) to usual health facility:**								
<30	167	39.2		–	–			
30–59	54	12.6		–	–			
60+	206	48.3		–	–			
Median minutes	427	40.0	(10, 120)	–	–			

*Unless otherwise noted, comparison data are for surviving children who were their mother’s most recent birth in 2007–2010 and for their families from the 2010 Niger National Mortality Survey.

**†**VASA and comparison data are for ever–married women 20–49 years old at the time of the survey. The comparison data are from the 2012 Niger Demographic and Health Survey.

**Table 2:** Comparison of antenatal and delivery care indicators for 453 neonatal deaths with those of the general population, Niger, 2007–2010

	Neonatal deaths		Comparison data*		χ^2^	*P*–value
**Characteristics**	**N**	**%**	**N**	**%**		
**Antenatal care:†**						
At least 1 visit	336	74.1	3269	83.7	25.7	<0.001
4+ visits	140	31.6	1275	32.6	0.2	0.671
**Antenatal care content:†**						
Blood pressure	249	75.1	2432	74.4	0.1	0.81
Urine test	86	25.7	1252	38.3	20.8	<0.001
Blood test	129	38.7	1560	47.7	9.8	0.002
Danger sign counseling	117	35.1	1882	57.6	61.8	<0.001
Quality antenatal care (blood pressure, urine& blood test, counseling)	40	12.0	801	24.6	26.7	<0.001
Tetanus vaccination	283	62.4	4209	65.7	2.0	0.162
Antimalarial†	233	51.5	2760	70.6	69.3	<0.001
**Delivery place:**						
Hospital	26	5.9	113	1.8	36.1	<0.001
Other formal provider	101	22.3	2294	35.8	33.9	<0.001
Institutional delivery	127	28.1	2407	37.6	16.6	<0.001
En route to provider	12	2.6	–	–		
Home	313	69.1	3940	61.5	10.4	0.001
Other	1	0.2	57	0.9		
**Birth attendant:**						
Skilled	129	28.5	2148	33.5	4.8	0.028
Traditional birth attendant	148	32.7	3096	48.3		
Mother herself	92	20.4	–	–		
Other	84	18.5	1160	18.1		
**Delivery mode:**						
C–Section†	11	2.4	73	1.9	0.6	0.443

*Unless otherwise noted, comparison data are for mothers of surviving children who were their mother’s most recent birth in the 12 months prior to the 2010 Niger National Mortality Survey.

†Comparison data are for mothers of surviving children who were their mother’s most recent birth in 2007–2010 from the 2012 Niger Demographic and Health Survey.

**Table 3:** Comparison of maternal complications among early neonatal deaths in Niger, 2007–2010, with those of all births in Niger and perinatal and early neonatal deaths and neonatal survivors in other countries

		Multi–country survey* [12]								
	Niger VASA	Niger	All countries		Kenya [8]		Bangladesh [9]		Palestine [10]	
**ENM**	**All births**	**ENM**	**Surv**	**PNM**	**Surv**	**PNM**	**Surv**	**PNM**	**Surv**
**N = 298**	**N = 10 871**	**N = 2528**	**N = 298 912**	**N = 108**	**N = 802**	**N = 86**	**N = 1498**	**N = 80**	**N = 808**
**%**	**%**	**%**	**%**	**%**	**%**	**%**	**%**	**%**	**%**
**Pregnancy complications:**										
Maternal infection†	17.9	0.2	2.6	0.5	–	–	–	–	–	–
Antepartum hemorrhage	12.5	0.5	5.7	0.6	8.3	0.4	12.8	2.7	12.5	0.7
Eclampsia/preeclampsia	9.5	1.6	9.7	2.2	–	–	19.8	8.6	26.3	18.4
Premature rupture of membranes	6.8	–	–	–	12.0	1.6	–	–	–	–
Malaria	6.7	–	0.5	0.1	–	–	–	–	–	–
Anemia	3.1	–	4.8	1.2	–	–	–	–	–	–
Diabetes	0.0	–	–	–	–	–	–	–	–	–
Any pregnancy complication	36.4	–	–	–	–	–	–	–	37.5	7.4
**Labor and delivery complications:**										
Intrapartum hemorrhage	16.1	–	1.4	0.2	–	–	–	–	–	–
Prolonged labor	5.2	–	–	–	17.6	7.2	–	–	7.5	4.3
Preterm labor	8.2	–	52.2	6.0	12.0	1.6	45.3	21.7	57.5	9.3
Maternal infection	4.5	–	–	–	–	–	–	–	–	–
Eclampsia/preeclampsia	0.8	–	–	–	–	–	–	–	–	–
Malaria	0.7	–	–	–	–	–	–	–	–	–
Anemia	0.3	–	–	–	–	–	–	–	–	–
Any labor/delivery complication	28.0	–	–	–	60.2	14.0	–	–	45.0	24.3
**Any maternal complication**	52.6	–	–	–	–	–	–	–	–	–

ENM – early neonatal mortality, PNM – perinatal mortality, Surv– neonatal survivors

*The referenced study did not distinguish between antepartum and intrapartum complications. The distinctions made here are based on information provided in the paper; for example, placenta previa was categorized as antepartum hemorrhage, and ruptured uterus as intrapartum hemorrhage.

†For Niger VASA ENM, maternal infection = sepsis; for all multi–country survey results, maternal infection = any one or more of pyelonephritis, influenza–like illness, other systemic infection/sepsis.

### Statistical analysis

This study was mainly descriptive. Percentages, means and medians are reported for demographic factors, causes and social determinants of neonatal deaths, maternal complications associated with the deaths, and the available comparison data. The χ^2^–test was used to assess differences between proportions for the VASA findings and comparison data. The VASA analysis was adjusted for sampling weights, taking into account the cluster design nature of the 2010 NMMS that identified the deaths. The 2010 NMMS and 2012 NDHS analyses that provided the comparison data also were adjusted for cluster sampling using the sampling weights for those studies.

### Ethical approval

The study was approved by the National Consultative Ethics Committee of the Niger Ministry of Health and the Institutional Review Board of the Johns Hopkins Bloomberg School of Public Health. Informed consent was given by all study participants prior to their being interviewed.

## RESULTS

Ninety–seven percent of the respondents for the 453 neonatal deaths were the mother of the deceased child. Nearly two–thirds (65.8%) of the deaths occurred during the first six days of life. More than half (62.9%) the newborns died from an infectious disease, nearly 20% succumbed to intrapartum-related complications (including 17.9% with birth asphyxia alone, 1.1% with signs of birth injury alone, and 0.9% with both birth asphyxia and injury), and 2.6% each to congenital malformations and complications of preterm delivery (**Figure 1**).

### Demographic factors

As shown in **Table 1**, the deceased neonates were significantly different from surviving children born in the same population during the same years for several demographic characteristics. There was a greater male predominance among the deaths than among surviving children (58.1% vs 50.8%, χ^2^ = 9.3, *P* = 0.002). More of the deceased neonates than the survivors were their mother’s first born child (25.3% vs 14.1%, χ^2^ = 44.3, *P* < 0.001). Their mothers also gave birth at a younger age—more of the decedents’ mothers were less than 20 years old, both at the time of the index child's birth (22.8% vs 15.7%, χ^2^ = 16.3, *P* < 0.001) and when they had their first birth (71.0% vs 66.2%, χ^2^ = 4.5, *P* = 0.034), than mothers of surviving children. In addition, more of the deceased children’s mothers had no formal education (86.6% vs 82.7%, χ^2^ = 4.7, *P* = 0.030). The households of the children also differed in that more families of the deceased than the survivors resided in a rural area (90.3% vs 81.8%, χ^2^ = 21.5, *P* < 0.001). Comparison data are lacking for travel time to the usual health facility used. Nevertheless, the data for the decedents reveals a long travel time (40 minutes). The mothers both of decedents and survivors first married when they were very young.

### Antenatal and delivery care

Fewer decedents’ than survivors’ mothers accessed any antenatal care (ANC) (74.1% vs 83.7%, χ^2^ = 25.7, *P* < 0.001), although only about 32% of both groups made at least the recommended four visits (**Table 2**). However, twice as many mothers of the survivors (24.6% vs 12.0%, χ^2^ = 26.7, *P* < 0.001) who made at least one ANC visit received quality care consisting of all of four key ANC interventions. The biggest gap in the individual ANC components was in counseling on the danger signs of pregnancy requiring urgent careseeking. Survivors’ mothers also were more likely to take a prophylactic anti–malarial during their pregnancy (70.6% vs 51.5%, χ^2^ = 69.3, *P* < 0.001), to have an institutional delivery (37.6% vs 28.1%, χ^2^ = 16.6, *P* < 0.001) and to be cared for by a skilled birth attendant (33.5% vs 28.5%, χ^2^ = 4.8, *P* = 0.028). Only about 2% of both groups were delivered by Caesarean section.

### Maternal complications and careseeking

**Table 3** shows that more than half (52.6%) of the 298 mothers with an early neonatal death had a serious pregnancy or labor and delivery complication, with the level for individual pregnancy complications ranging up to 17.9% for maternal infection and for individual labor and delivery complications up to 16.1% for intrapartum hemorrhage. While there were no comparison data for these findings in the NNMS or NDHS, a district hospital study in Kenya [8], community–based studies in Bangladesh [9] and Palestine [10], and a multi–country hospital–based survey [12] found comparable, some higher and some lower, levels of these same complications in women with a perinatal or early neonatal death and, by country, uniformly lower levels in women with a surviving neonate. The multi–country survey included Niger, which had much lower levels for three maternal complications among all births than found for the deaths in our study (**Table 3**).

Because early onset neonatal infection is common in newborns whose mothers have maternal infection or colonization [36], we also explored the relationship of maternal infection to early onset (at less than 2 complete days of life) neonatal infection (sepsis, meningitis or pneumonia) as the primary cause of neonatal death. We demonstrated a significant positive association between these maternal and neonatal conditions, both when comparing early onset to late onset neonatal infection (28/95 [30.0%] vs 17/146 [11.3%], χ^2^ = 13.2, *P* < 0.001) as well as to all other neonatal deaths from any cause (28/95 [30.0%] vs 65/358 [18.1%], χ^2^ = 6.5, *P* = 0.011) [23].

**Figure 2:**
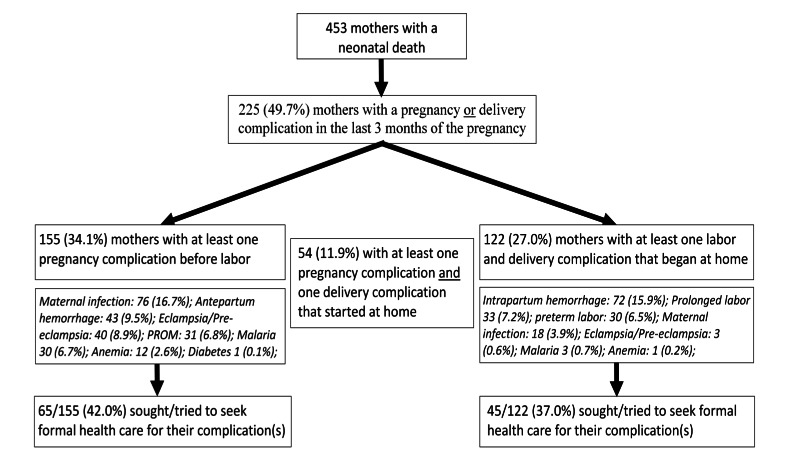
Maternal complications and careseeking during the pregnancy and delivery for 453 neonatal deaths, Niger, 2007–2010.

**Figure 2** shows the maternal complications and careseeking for these for all 453 women with a neonatal death. Fewer than half the women with a pregnancy complication (65/155, 42.0%) or labor and delivery complication that began at home (45/122, 37%) sought any formal health care for their complications, and they were no more likely to deliver at a health facility than women without a complication (any pregnancy complication: 32.7% vs 25.8% without, χ^2^ = 2.45, *P* = 0.118; any delivery complication: 25.1% vs 29.3% without, χ^2^ = 0.79, *P* = 0.373). Almost all this care was sought at the primary care level. Of the 45 women who sought or tried to seek formal care for a labor and delivery complication, 21 (46%) went to a CSI, 5 (12%) to a Case de Santé, 11 (24%) to a primary care facility of undetermined type, and 3 (7%) said that they saw a nurse or midwife in the community. Only four (10%) of the women first went and four (10%) more later went to a hospital. Eight (17%) delivered in a hospital, 20 (44%) in a CSI, Case de Santé or undetermined primary care facility, 3 (7%) on route to a facility and 14 (32%) at home. Of the labor and delivery complications that occurred in more than 3% of the women, formal health care was sought most often for maternal sepsis (9/18 women, 51.7%), followed by preterm labor (14/30, 48.6%), prolonged labor (14/33, 42.9%) and intrapartum hemorrhage (26/72, 35.8%). However, as for all delivery complications, women with these complications were no more likely to deliver at a health facility than women without a delivery complication.

### Normal newborn care

Almost a third (144, 31.7%) of the babies that died were bathed within one hour after birth, and 351 (79.1%) were bathed before 24 hours after birth, which is the recommended lower time limit for first bathing [37]. An appropriate measure was taken to keep 406 (90.0%) of 451 newborns warm after birth, but only 42 (9.9%) of 423 were breastfed in the first hour after birth, compared to 2350 (42.9%) of 5478 surviving children born in the prior 24 months identified by the 2010 NNMS (χ^2^ = 177.1, *P* < 0.001). In all, only 1 of 408 deceased neonates received quality postnatal care in the first day of life (ie, sterile blade used to cut the cord, baby not bathed in the first 24 hours after birth, baby dried and wiped or wrapped in a blanket or given skin to skin contact or placed in an incubator after birth, and baby breastfed within 1 hour after birth).

### Failures in the pathway to survival

**Figure 3:**
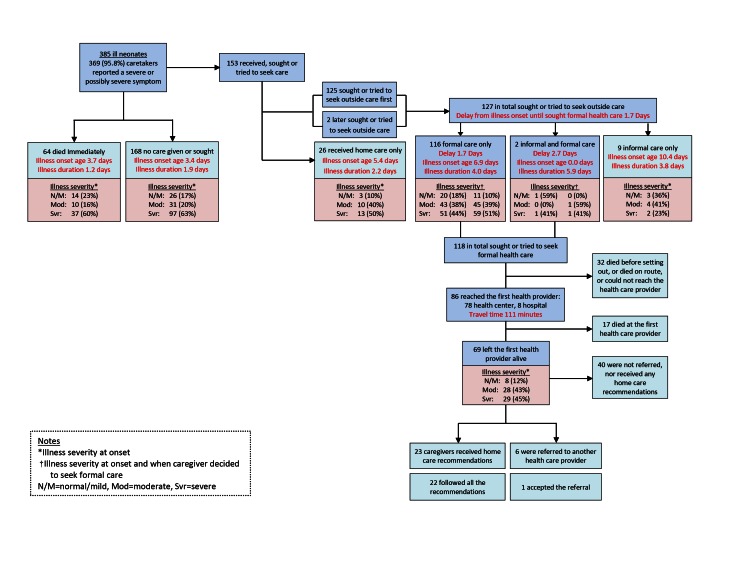
The “Pathway to Survival” for 385 neonatal deaths born at home or left the delivery facility alive, Niger 2007–2010.

**Figure 3** illustrates the careseeking process from home for the 385 deceased neonates who either were born at home or delivered at a health facility and left alive. Although nearly all (95.8%) caregivers reported that the first symptom of their child’s illness was either a severe or possibly severe symptom, 232 (60.3%) neonates, 64 of whom were said to have died “immediately,” received no care for their fatal illness.

The mean age at illness onset for the 232 newborns who received no care was 3.5 days and their mean illness duration was 1.7 days, compared to illness onset at age 6.8 days and duration of 3.7 days for the 153 (39.7%) neonates whose caregiver’s first action was to provide home care or seek care outside the home. Caregivers’ reports of their child’s illness severity as rated according to their feeding behavior, alertness and activity level confirmed that more of the 232 newborns who received no care were severely ill at the start of their illness than of the 153 newborns who received some care (62.4%, vs 44.0%; χ^2^ = 11.6, *P* < 0.001), corresponding with the earlier onset and more rapid progression of their illnesses.

**Figure 4:**
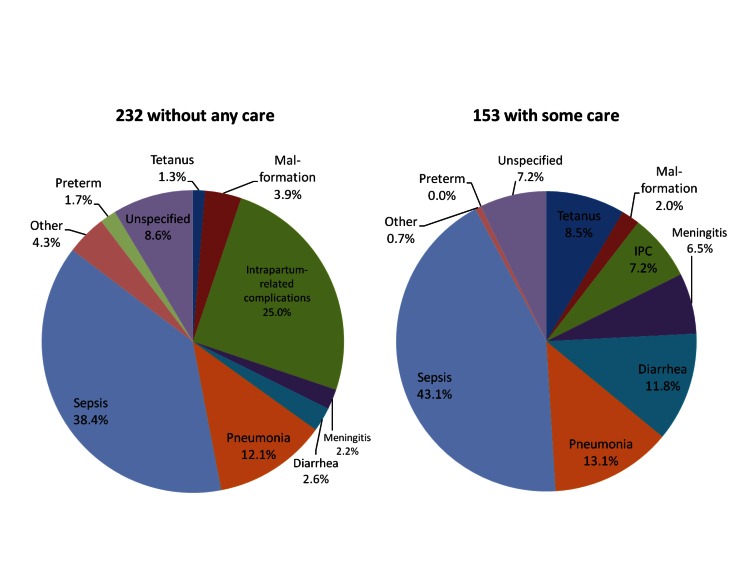
Expert algorithm, hierarchical verbal autopsy causes of death for 385 neonates born at home or left the delivery facility alive, with and without any care given or sought from home, Niger, 2007–2010.

The causes of death of the two groups (**Figure 4**) also reflected their age and illness course, with more deaths due to birth asphyxia and preterm delivery in the younger group with faster illness progression and no care provided or sought than among the older neonates who received care (26.7% vs 7.2%), and more infectious deaths in the older group with longer illnesses and some care than among the younger neonates without care (83.0% vs 56.6%). Of note is that 8.5% of the 153 late neonatal deaths that received some care were due to tetanus.

**Figure 5:**
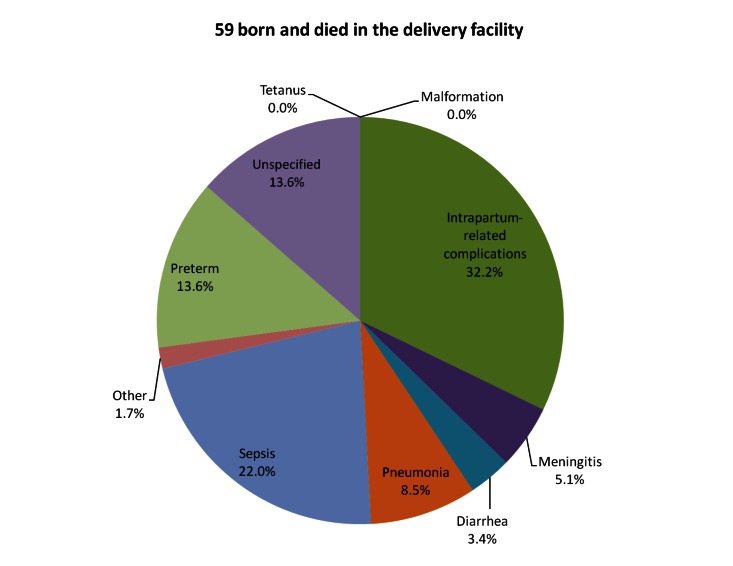
Expert algorithm, hierarchical verbal autopsy causes of death for 59 neonates born and died in the delivery facility, Niger, 2007-2010.

For comparison, **Figure 5** shows the causes of death of 59 neonates not included in **Figure 3** because they died in their delivery facility without ever leaving. The cause distribution was more similar to that of the 232 neonates who died at home without any care given or sought, though even more skewed toward birth complications and preterm delivery over infectious causes (45.8% vs 39.0%), while their mean age at illness onset (1.0 days) was younger and their illness duration (2.3 days) was somewhat longer. Most remarkable was that eight of the 12 deaths due to preterm delivery in all 453 neonates occurred among the 59 babies that were born and died in a health facility without leaving. Of the other four preterm deaths (all among the 232 newborns that died at home without seeking care), three were delivered in a health facility and one at home.

**Figure 3** also shows that formal health careseeking was attempted for only 118 of the 153 neonates who received any care. Among these 153 newborns, there was no difference in formal careseeking for males and females (78.2% vs 76.6%, χ^2^ = 0.5, *P* = 0.816). Formal careseeking was delayed for 1.7 to 2.7 days, nearly half way into the illness course, by which time half the children were severely ill. Though the children for whom formal care was sought were less severely ill at the onset of their illness and their illnesses progressed more slowly than those of the children who received no care, the delay in careseeking was associated with 32 (27.1%) of the 118 neonates dying before reaching the formal provider. However, there was no significant difference in the proportions of newborns whose illness severity increased between those who reached and did not reach the provider (20.2% vs 9.6%, χ^2^ = 1.76, *P* = 0.416), and equal proportions were severely ill at the time the decision was taken to seek formal care (48.4% vs 55.8%, χ^2^ = 0.51, *P* = 0.47).

Almost all formal care was sought at lower level health facilities; only eight (6.8%) of the 118 newborns initially went to a hospital, only six (8.7%) of 69 who left a first level facility alive were referred, and only one of the six accepted the referral. The most common actions taken by the first health facilities reached by the 86 neonates included: 34 (39.9%) were given an intramuscular medication, 20 (23.7%) received an oral anti–malarial, 18 (20.7%) received another oral medicine, and 13 (15.6%) were given an oral antibiotic. On average, each neonate received 1.7 treatments; the health provider did “nothing” for only 6 (7.0%) neonates. One–third of the 69 caregivers of neonates discharged alive from a first level facility received recommendations for home care, and all but one of those were able to follow all the recommendations. **Figure 3** also shows that 29 (45%) of the 69 children that left the first provider alive were still rated by their caregiver as being severely ill at discharge.

Few comparison data for surviving children in Niger are available for these careseeking findings. The 2010 NNMS asked about formal careseeking for children with a fever or cough in the two weeks prior to the survey. For fever, 71.6% of 6 neonates, 56.5% of 20 1–month olds, and 62.8% of 71 2–month olds sought formal care, for a total of 62.8% of 97 children under 3 months of age; while for cough, all 3 neonates, 8.5% of 8 1–month olds, and 52.7% of 49 2–month olds sought formal care, for a total of 48.5% of 60 children under 3 months of age. These figures compare with the 118 (30.6%) and 86 (22.3%) of 385 neonates with a fatal illness who, respectively, sought formal health care and reached the first formal provider. Limiting the comparison to the 151 deceased neonates who survived at least one week, which is closer to the age distribution of the surviving children, 73 (48.3%) and 54 (35.7%), respectively, sought formal health care and reached the first provider.

### Constraining factors for maternal and newborn careseeking

Concerns of caregivers that contributed to delays in careseeking were similar for pregnancy complications, delivering at a health facility and newborn illnesses, with some notable exceptions (**Table 4**). Most women, even those who did not seek formal health care, said they had no concerns that kept them from seeking care, although more of those who did not seek care reported one or more concerns. Of 90 women who did not seek formal care for their pregnancy complication(s), 26 (28.7%) reported, on average, 2.0 constraints each. The most common concerns were the cost for transportation or health care (13), distance to a facility (12) and the lack of transportation (9). Cost and distance also were the main constraints for those who sought formal care, but only 7 (10.8%) of the 65 women who sought care reported that they had any careseeking constraints. Similarly, distance and transportation were the main constraints women had for delivering at a health facility and seeking care for their newborn’s illness, with cost being a lesser issue for both. Unlike for the other two situations, underestimating illness severity was an often stated constraint to careseeking for newborn illnesses. However, an incongruity to be considered is that 7 of the 17 caregivers who reported that their baby was not sick enough to need health care also ranked the child as being severely ill.

**Table 4:** Constraints for formal health careseeking for three situations contributing to neonatal deaths, 2007–2010, Niger

	Pregnancy complications				Health facility delivery				Newborn illness			
	**65 sought formal care**		**90 did not seek formal care**		**127 delivered at a facility**		**326 did not deliver at a facility**		**118 sought formal care**		**202 did not seek formal care**	
	**N**	**%**	**N**	**%**	**N**	**%**	**N**	**%**	**N**	**%**	**N**	**%**
**Constraints:**												
Did not think she/the baby was sick enough to need health care	0	0	2	1.9	0	0.2	4	1.1	0	0	17	8.3
No one available to go with her	0	0	1	1.3	0	0.2	8	2.4	0	0	1	0.3
Too much time from her regular duties	1	1.7	1	1.2	0	0.2	2	0.7	0	0	0	0
Someone else had to decide	0	0	6	6.6	0	0	3	0.9	0	0	4	2.1
Too far to travel	3	4.7	12	12.9	2	1.3	49	15.0	10	8.5	18	8.8
No transportation available	1	1.7	9	10.4	3	2.4	43	13.1	12	10.2	17	8.4
Cost (transport, health care, other)	3	5.1	13	14.4	5	3.9	17	5.3	4	3.0	6	3.0
Not satisfied with available health care	2	2.4	1	1.6	0	0	7	2.1	2	1.4	2	1.2
Symptom(s) required traditional care	0	0	3	2.8	0	0	0	0	1	0.7	2	0.8
Thought she/baby was too sick to travel	0	0	0	0	0	0	2	0.7	0	0	3	1.5
Thought she/baby will die despite care	0	0	0	0	0	0	0	0	0	0	6	2.9
Was late at night (transportation or provider not available)	–	–	–	–	1	0.6	5	1.5	0	0	1	0.4
Fears exposure to male health provider	0	0	1	0.8	0	0	2	0.7	–	–	–	–
Other	0	0	4	4.0	1	1.1	21	6.3	1	1.0	3	1.3
**Total careseekers:**	6	8.8	26	28.7	7	5.7	89	27.3	16	13.6	44	21.8
**Total constraints:**	10		52		12		163		30		80	

## DISCUSSION

We undertook a verbal/social autopsy study of recent neonatal deaths in Niger to examine critical factors that might help explain the non–decrease in neonatal mortality in light of the significant decline in child mortality from 1998 to 2009. The study deaths were identified by the 2010 NNMS, the same survey used to establish the mortality trends [2], and that provided most of the data on surviving children and their families that we compared to the deaths. We determined the cause distribution of the deaths and related maternal complications, as these can highlight needed interventions, and focused on social, behavioral and health system determinants that influence the strategies required to effectively deliver maternal and child survival interventions [13].

### Demographic factors, normal maternal and newborn care

The examination of demographic factors revealed several significant differences between deaths and survivors of the same cohort, with all factors in the direction of risk for the deaths. Some of these are potentially modifiable in the long term, while knowledge of some others might help in targeting interventions.

The excess in rural residence of deceased neonates, combined with their long travel time to the usual health facility, suggests that some remote communities remain at risk due to limited access to primary care despite recent efforts in Niger that have brought a package of high–impact interventions at integrated health centers and posts to within 5 km of 80% of the population [38].

The predominance of deaths of male neonates agrees with a well–established pattern of excess male mortality that suggests this is due to an unmodifiable, biological effect [39,40]. On the other hand, the excess in neonatal deaths of firstborns, among women under age 20 and women with no formal education, suggests the need to provide young women and new mothers with information and support to better care for themselves and their newborn children. A community approach is necessary to accomplish this goal [41]. Ensuring universal access to four high quality antenatal care visits, including counseling on maternal health, normal newborn care, and pregnancy, delivery and newborn danger signs, also could help achieve this aim. The low median age at first marriage and low levels of formal education and antenatal care for mothers of surviving as well as deceased neonates shows that much work remains to be done to improve these indicators, and that encouraging girls and young women to delay marriage and instead go to school might help achieve these objectives.

Particular essential antenatal and delivery interventions that remain at low levels in the general population of Niger were found to be at very low levels among the mothers of deceased neonates, including taking a prophylactic anti–malarial medication during pregnancy, institutional delivery and attendance at birth by a skilled person. These finding suggest the need to expand the scope of the essential care package provided at integrated health centers and posts to better cover the needs of pregnant women as well as the accessibility of facilities.

The very low level of breastfeeding within one hour of birth found for the newborns who died, one–fourth of the general population’s 43%, might have been partly due to sick newborns being unable to feed. However, the mean age at illness onset of 1.3 days for the two–thirds of neonates that died in the first week of life and the odds of not being breastfed within one hour of birth for neonates with illness onset at age 0 days vs 1 to 6 days (2.03, 95% CI = 0.87, 4.73), argues that this was not a major factor. There was no comparison data for other normal newborn care measures, but the low levels for deceased neonates, summarized as the 1 of 408 newborns that received quality postnatal care in the first day of life, suggest that these factors contributed to the deaths. As with many of the maternal care indicators for which there was comparison data, the very low levels for the newborn care indicators among the deaths appear to be the extreme of overall low population levels that have helped maintain the high neonatal mortality rate in Niger. This is supported by the comparison of coverage data for newborn care indicators that are available both for Niger and other sub–Saharan Africa (SSA) countries, for postnatal care of all neonates within 2 days of birth (Niger, 13% vs 35% for 15 other SSA countries) and for registration of live births in 2012 by age 1 year (Niger, 30% vs 50% for 44 other SSA countries) [42].

### Maternal complications and careseeking

Multiple studies conducted in low and middle income countries have found that maternal complications constitute the greatest risks for perinatal and early neonatal mortality [8–12]. Two–thirds of the neonatal deaths in our study occurred in the first week of life, and half the women with an early neonatal death had a pregnancy or labor and delivery complication. The much lower levels of maternal infection, antepartum hemorrhage and preeclampsia/eclampsia in mothers with a surviving neonate in Niger [12] and the generally far lower levels for these and other complications in women with a surviving neonate found by other studies [8–10] strongly suggest that the high rate of maternal complications identified by the VASA study significantly contributed to the neonatal deaths. This conclusion is supported by the positive relationship we identified between maternal infection and early onset severe neonatal infection as the primary cause of neonatal death. We are unaware of any prior study that has demonstrated this association at community level using verbal autopsy methodology.

Fewer than half the women with a pregnancy or labor and delivery complication sought formal health care. The most common reasons stated for not seeking care for a pregnancy complication, as well as for not delivering at a health facility, were distance to the facility, the lack of transportation and cost. However, the most striking finding was that nearly three–fourths of the women who did not seek care or deliver at a facility could not state a constraint to their use of the facility. This points to the need for further research to better understand this phenomenon, as well as the need to bring the required care to, or closer to, the community and to strengthen the links between communities and community–based providers with the health system. Recent studies have examined and shown promise for several potential strategies, including increased referrals to health facility for pregnancy related complications [43], community mobilization to increase institutional births, financial incentive plans and community referral/transport systems to increase rates of skilled birth attendance and the use of emergency obstetric care [14]; but it is not yet clear whether skilled birth attendance can be successfully provided in the community [15]. To help monitor such programs, periodic national surveys such as the DHS should consider adding questions on maternal complications, careseeking for these, and women’s success in receiving care for these important risk factors for maternal and perinatal morbidity and mortality.

### Failures in the pathway to survival

While almost all caregivers reported one or more signs of severe illness at the onset of their newborn’s sickness, less than half sought or provided care. This decision appears to have been influenced by caregivers’ perception of their child’s illness severity, rather than their recognition of severe illness signs. We included feeding behavior, alertness and activity level in ranking perceived illness severity, as these are signs that caregivers both recognize and perceive to indicate the need to seek formal health care for sick neonates [29–32] and, indeed, we found that careseeking was associated with these illness signs. The lower level of careseeking for the most severely ill newborns with early onset illness supports the hypothesis that caregivers believed they could not take any effective action for these children. Alternatively, their lack of action might have been due to there being less time to provide care before the children died.

In a multivariable analysis of careseeking for fatal newborn illnesses in Bangladesh that accounted for the competing risk of dying before careseeking, we also found that caregivers’ reporting that their child was “not moving” at illness onset did not positively affect careseeking, while a child that was “less active than normal” was more likely to be taken for formal care [44], lending support to the severity perception theory. While several newborn illness danger signs are included in the WHO/UNICEF IMCI algorithm [29], it would appear to be more efficient and effective for careseeking messages at household and community level to focus on the signs that are most intrinsically perceived as indicating the need to seek care. The incongruity that we found between the illness severity ranks and some caregivers’ apparent underestimate of their child’s illness severity suggests that there is a need to provide caregivers with information even on the more well–perceived signs.

For the children whose illnesses began after the early neonatal period, in the aggregate careseeking was delayed until their illnesses had progressed from mild or moderate to severe. This may have contributed to the deaths, although this conclusion is tempered by the finding of equal severity among those who reached and did not reach the first health provider alive. Other studies both of fatal and nonfatal child illnesses have found that first careseeking to an informal provider contributed to delays in formal careseeking, which was undertaken only after the child’s illness progressed to a severe state [31,33,45]. This was not an important factor in the current study, in which both informal and formal careseeking were sought for only two newborns and nine others received only informal care.

Illness severity was related to younger age of the child at illness onset and to faster illness progression, which in turn were related to the cause of death. While infectious diseases caused 60% of the neonatal deaths overall, and birth asphyxia and preterm delivery together accounted for another 27%, the distributions of these causes varied by age, place of death and care provided for the fatal illness. Birth asphyxia and preterm delivery were more common causes of early neonatal illnesses and deaths of children who died in the facility of their birth without leaving or at home without receiving or seeking care, compared to the predominance of infectious causes of the late neonatal illnesses and deaths of children who received home care or sought care from home. Yet, infections also were common causes of early neonatal deaths in facilities and caused twice as many early neonatal deaths in the community as did birth asphyxia.

The more similar cause distributions of neonates who died in their birth facility without leaving and at home without care suggests that the causes of death were as or more related to the children’s ages than to any care they received or did not receive, though the still greater proportion of early neonatal infectious deaths in the community suggests that facilities may have had somewhat more success in treating infectious causes than intrapartum–related events and preterm birth complications. A facility–based study of presenting illnesses, treatments provided, quality of care, case fatality and cause–specific mortality rates would be needed to accurately determine the impact of facility birth on mortality and its cause distribution. The excess deaths from birth asphyxia in both settings and from preterm delivery in facilities, combined with the finding that women with delivery complications were equally likely to deliver at home as in a facility, suggests the need to improve the quality of intrapartum care to decrease deaths from these causes in the community and in health facilities. This strategy, along with postnatal care such as exclusive breastfeeding and providing antibiotics to reduce deaths from infectious causes, has been shown to provide the greatest reduction in neonatal mortality [13].

Fully 83% of the late neonatal deaths, that is, the deaths for which most affected children received some formal health care, were due to infectious causes. The limited comparison data available for these children suggest that young infants in Niger with a non–fatal fever or cough are taken for health care as or more often than severely ill late neonates. This corresponds with our finding that neonates who were moderately ill at illness onset were more likely to be taken for care than those who were severely ill. However, the decedents’ younger age and the inability to match deaths with survivors on illness severity and the timing of careseeking underscores the difficulty in identifying an appropriate comparison group for examining careseeking in fatal illnesses. The finding that 8.5% of the late neonatal deaths were due to tetanus deserves further investigation that is beyond the scope of the current study.

As discussed for the early neonatal deaths, accurately assessing the quality of care provided in health facilities for the late neonatal deaths would require conducting a facility–based study. However, the VASA study offered some indication of problems that point to the need for just that. Caregivers reported that nearly one–fourth of the 86 neonates treated in a health facility were given an anti–malarial medication. If correct, that would most likely be a treatment error, as such young children would be much more likely to have had bacterial sepsis than malaria [46]. The fact that caregivers rated 45% of the 69 children that left the first health provider alive as severely ill, while only 9% of these children were referred, is another indicator of a potentially serious deficit in the quality of care provided at these facilities.

### Study limitations

The VASA study was not designed to examine changes in any factors over time and so cannot strictly conclude that a lack of change in the examined factors led to the non–decrease in mortality. However, the study provides plausible evidence by identifying low levels among the decedents of factors known to be critical to neonatal well–being and survival, and for many of these factors by showing that their levels in the decedents were significantly below those of surviving newborns from the same cohort. Also, the VASA study was retrospective in design, with an average recall period of 3.5 years. This could lead to inaccuracies in respondents’ recall of events. Yet, the recall period for the survivors data examined for this analysis was similar to that of the decedents, which should minimize any recall bias in comparisons of findings for the decedents and survivors.

In conclusion, the VASA study revealed multiple factors contributing to the non–decrease in neonatal mortality that can be most effectively tackled through an integrated maternal–neonatal care package in the community and at health facilities. The predominance of rural residence and the role of distance and transport constraints to reaching a facility point to the need for Niger to scale up its recently implemented package of high–impact interventions to additional integrated health centers and posts. The low level of quality antenatal care and skilled birth attendance, high level of maternal complications, and many deaths from birth asphyxia and early onset severe neonatal infection in the community and health facilities call for expanding the package to provide antenatal and intrapartum care, with support for reaching a higher level facility when needed; while community education, mobilization and support are needed to improve illness recognition and careseeking for early and late onset severe neonatal infection. The quality of intrapartum and neonatal infectious disease care in first–level facilities and hospitals should be assessed and, if found to be required, improved.

Box 1 **Definitions of maternal complications**

**Pregnancy complications**

Antepartum hemorrhage: Any vaginal bleeding before the onset of labor

Preeclampsia/eclampsia: (Puffy face and (blurred vision or severe headache or high blood pressure)) and/or (Convulsions and no fever and no history of convulsions)

Maternal infection: Fever and (severe abdominal pain or smelly vaginal discharge)

Maternal anemia: (Severe anemia or (pallor and shortness of breath)) and (too weak to get out of bed or fast or difficult breathing)

Gestational diabetes: Diabetes that started during pregnancy and before labor began

Premature rupture of the membranes: Water broke 6 hrs or more before labor began

Malaria: Convulsions and fever

**Labor/delivery complications (start after labor onset)**

Intrapartum hemorrhage: Excessive bleeding during labor or delivery

Preeclampsia/eclampsia: same as for pregnancy

Maternal infection: Fever and (severe abdominal pain or smelly vaginal discharge or foul smelling amniotic fluid)

Maternal anemia: same as for pregnancy

Preterm delivery: Less than 9 months

Prolonged labor: Labor for 12 or more hours

Malaria: same as for pregnancy
